# Neural Correlates of Fear of Movement in Patients with Chronic Low Back Pain vs. Pain-Free Individuals

**DOI:** 10.3389/fnhum.2016.00386

**Published:** 2016-07-26

**Authors:** Michael L. Meier, Philipp Stämpfli, Andrea Vrana, Barry K. Humphreys, Erich Seifritz, Sabina Hotz-Boendermaker

**Affiliations:** ^1^Interdisciplinary Spinal Pain Research (ISR), Chiropractic Medicine, Balgrist University HospitalZurich, Switzerland; ^2^Center of Dental Medicine, University of ZurichZurich, Switzerland; ^3^Department of Psychiatry, Psychotherapy and Psychosomatics, Hospital of Psychiatry, University of ZurichZurich, Switzerland; ^4^MR-Center of the Psychiatric Hospital and the Department of Child and Adolescent Psychiatry, University of ZurichZurich, Switzerland

**Keywords:** amygdala, chronic low back pain, fear of movement, fMRI, PPI, Kinesiophobia, insula, pain-related fear

## Abstract

Fear of movement (FOM) can be acquired by a direct aversive experience such as pain or by social learning through observation and instruction. Excessive FOM results in heightened disability and is an obstacle for recovery from acute, subacute, and chronic low back pain (cLBP). FOM has further been identified as a significant explanatory factor in the Fear Avoidance (FA) model of cLBP that describes how individuals experiencing acute back pain may become trapped into a vicious circle of chronic disability and suffering. Despite a wealth of evidence emphasizing the importance of FOM in cLBP, to date, no related neural correlates in patients were found and this therefore has initiated a debate about the precise contribution of fear in the FA model. In the current fMRI study, we applied a novel approach encompassing: (1) video clips of potentially harmful activities for the back as FOM inducing stimuli; and (2) the assessment of FOM in both, cLBP patients (*N* = 20) and age- and gender-matched pain-free subjects (*N* = 20). Derived from the FA model, we hypothesized that FOM differentially affects brain regions involved in fear processing in patients with cLBP compared to pain-free individuals due to the recurrent pain and subsequent avoidance behavior. The results of the whole brain voxel-wise regression analysis revealed that: (1) FOM positively correlated with brain activity in fear-related brain regions such as the amygdala and the insula; and (2) differential effects of FOM between patients with cLBP and pain-free subjects were found in the extended amygdala and in its connectivity to the anterior insula. Current findings support the FOM component of the FA model in cLBP.

## Introduction

Most individuals suffering from acute low back pain (LBP) recover within 6 weeks (Koes et al., 2001). However, a small minority develop disabling persistent and/or recurrent chronic LBP (cLBP) that accounts for a considerable burden in terms of pain and suffering, loss of productivity and substantial health care expenditures (Bronfort et al., 2004; Rapoport et al., 2004; Peterson et al., 2012). Fear of movement (FOM) has been increasingly recognized as a significant explanatory factor for developing cLBP (Vlaeyen and Linton, 2000; Buchbinder et al., 2001, 2008; Chou and Shekelle, 2010; Wertli et al., 2014). Derived from the Fear Avoidance (FA) model, the development of FOM is characterized by a vicious circle of various cognitive and behavioral aspects such as pain catastrophizing and avoidance behavior that may ultimately lead to physical deconditioning of the musculoskeletal system (Vlaeyen and Linton, 2000; Leeuw et al., 2007; Barke et al., 2012). In addition to pain as an unconditioned stimulus, FOM can also be triggered by fear inducing information and vicarious exposure, such as observations, regardless of the presence and intensity of back pain (Buchbinder et al., 2001, 2008; Buer and Linton, 2002; Gross et al., 2006; Meier et al., 2015). Nevertheless, excessive FOM results in heightened disability and is an obstacle for recovery from acute, subacute, and cLBP (Rainville et al., 2011). Despite a wealth of evidence that supports FOM as a strong predictor of disability in cLBP its underlying brain mechanisms remain unknown. So far, the only fMRI study involving cLBP patients with low and high FOM, measured by means of the Tampa Scale of Kinesiophobia (TSK) questionnaire, revealed no differential effects in fear-related brain activity between low and high FOM individuals. In support of these null results, a further study that investigated chronic musculoskeletal pain patients was not able to demonstrate a potential relationship between the TSK score and brain activity (Taylor et al., 2015). As such, the contribution of fear in the FA model along with the methodology to elicit FOM remained ambiguous (Barke et al., 2012; Salomons and Davis, 2012). However by using different FOM inducing stimuli, namely video clips of potentially harmful movements for the back, we recently have demonstrated that the FOM severity already has a neural impact in pain-free individuals by demonstrating a significant association between back pain-related FOM and brain responses in the left amygdala (Meier et al., 2015). As a consequence, the aim of the current fMRI study was to present these video clips to age- and gender-matched cLBP patients to investigate possible differential neural fear processing compared to pain-free subjects as a function of FOM that was assessed in both groups. Derived from the FA model, we hypothesized that FOM differentially affects brain regions involved in fear processing in patients with cLBP patients compared to pain-free individuals due to the recurrent pain and subsequent avoidance behavior in patients with cLBP.

## Materials and Methods

### Subject Recruitment and Questionnaires

The subjects were recruited via local chiropractic and physiotherapy centers and online advertisements. All subjects provided written informed consent for the participation in the experiment. The study was approved by the Ethics Committee Zurich and was conducted in accordance with the Declaration of Helsinki. The study sample consisted of 20 cLBP patients suffering from non-specific LBP (7 females, mean age = 39.35, SD = 13.97) and 20 pain-free subjects (12 females, mean age = 32.10, SD = 10.78). Inclusion criteria for cLBP was low back pain of at least 6 months duration. Exclusion criteria for cLBP patients were specific causes for the pain (ruled out by the chiropractor and/or manual therapist) and a history of psychiatric or neurological disorders. Exclusion criteria for the pain-free subjects were acute and/or recurrent back pain within the last 6 months, past chronic pain episodes, and a history of psychiatric or neurological disorders. The groups were age- and gender-matched (Table 1).

**Table 1:** **Participants characteristics**.

	cLBP patients (*n* = 20, mean (±SD))		Pain-free controls (*n* = 20, mean (±SD))	
	Low FOM (*n* = 10)	High FOM (*n* = 10)	Low FOM (*n* = 10)	High FOM (*n* = 10)	Statistical test**
Age	42.2 (12.2)	36.5 (15.6)	36.5 (13.6)	27.7 (4.2)	ns*
Gender	4 females	3 females	5 females	7 females	ns
Ratings HM^1^	5.40 (2.7)	5.46 (2.1)	6.00 (1.98)	4.25 (2.18)	ns
Rating NM^2^	0.84 (0.65)	1.71 (1.69)	1.67 (1.74)	0.78 (0.72)	ns
TSK(-G)	33.2 (3.9)	40.6 (4.5)	30.2 (4.1)	40.8 (6.0)	–
STAI state	41.3 (4.6)	46.1 (3.7)	42.3 (3.36)	42.9 (4.5)	ns
STAI trait	40.5 (4.4)	45.5 (6.6)	41.8 (2.78)	43.7 (3.3)	ns
FABQ	28.7 (16.5)	42.2 (26.41)			ns
Average pain	4.1 (2.0)	3.4 (1.8)			ns
Max pain	6.4 (1.8)	5.9 (2.5)			ns
Current pain	3.4 (2.3)	4.15 (2.7)			ns
Bournemouth	20.2 (11.3)	28.4 (13.42)			ns

^1^Harmful movements, ^2^neutral movements, *ns, not significant (*p* > 0.05), **see “Subject Recruitment and Questionnaires” Section. FOM, fear of movement; cLBP, chronic low back pain; TSK(-G), German version of tampa sacle of kinesiophobia; FABQ, Fear-Avoidance Beliefs Questionnaire.

Following an online advertisement, both groups completed the TSK questionnaire to assess the level of FOM (Kori et al., 1990). In the cLBP group the FOM severity was measured by using the 17-item German version of the TSK which has a satisfactory internal consistency (Cronbach’s *α* = 0.76–0.84) and contains statements focusing on fear of physical activity which were rated by the participants on a 4-point Likert scale from 1 = “strongly disagree” to 4 = “strongly agree” (Barke et al., 2012; Rusu et al., 2014). In addition, the cLBP patients completed the painDETECT questionnaire that included three 11-point numeric rating scales (NRS), with 0 being “no pain” and 10 being the “worst imaginable pain”. The triple NRS scales measure current pain, strongest and average pain intensity in the previous 4 weeks (Freynhagen et al., 2006). To further assess LBP complaints and concomitant psychosocial factors, patients filled in the Bournemouth questionnaire (Bolton and Breen, 1999). The Bournemouth questionnaire is a short, self-report questionnaire using visual analog scales (VAS) to assess the seven core items of LBP developed from the biopsychosocial model (i.e., pain intensity, function of activities of daily living and social activities, anxiety, depression levels, FA behavior, and locus of control). Finally, FA beliefs in cLBP patients were assessed using the German version of the Fear-avoidance Beliefs Questionnaire (FABQ-D; Pfingsten et al., 2000). The FABQ-D consists of 16 items where participants had to rate their agreement on a 7-point rating scale (0 = “completely disagree” to 6 = “completely agree”).

The pain-free group completed a modified 17-item German version of the TSK (TSK-G; Houben et al., 2005). The TSK-G also consists of a 4-point Likert scale ranging from 1 = “strongly disagree” to 4 = “strongly agree” and included questions such as “If I had pain and I were to try to overcome it, my pain would increase”. The questionnaire was originally validated in a Dutch sample of 2240 individuals divided in two groups of people with and without back complaints. Psychometric research indicated a sufficient reliability (Cronbach’s *α* = 0.78) and high TSK scores predicted pain catastrophizing, pain intensity and pain-related health indices. Therefore, the authors recommended the use of the TSK-G as a measure of FOM in general population studies (Houben et al., 2005). Furthermore, all subjects completed the State and Trait Anxiety Inventory (STAI) which is a common questionnaire that measures state and trait anxiety levels (Spielberger, 1971).

The questionnaire data was analyzed by dividing both groups into low- and high-FOM individuals using a median split (pain-free group median = 35, cLBP group median = 37.5) that resulted in four groups with 10 subjects, respectively. One-way ANOVAS (for gender: Pearson Chi-Square test) were conducted for questionnaire data that existed over all four groups whereas independent two-sample *t*-tests (two-tailed) were performed for data that applied only to the cLBP group (Table 1).

### Scanning Parameters

All measurements were performed on a 3-T whole-body MRI system (Philips Achieva, Best, Netherlands), equipped with a 32-element receiving head coil and MultiTransmit parallel RF transmission. Each imaging session consisted of a survey scan, a B1 calibration scan (for MultiTransmit), a SENSE reference scan and a high resolution T1-weighted anatomical scan. fMRI data were acquired with whole-brain gradient-echo echo planar imaging (EPI) sequences (365 volumes), consisting of 37 slices in the axial direction with the following parameters: field of view (FOV) = 240 × 240 mm^2^; acquisition matrix = 96 × 96; slice thickness = 2.8 mm; interleaved slice acquisition; no slice gap; repetition time (TR) = 2100 ms; echo time (TE) = 30 ms; SENSE factor = 2.5; flip angle 80°. Anatomical data were obtained with a 3D T1-weighted turbo field echo scan consisting of 145 slices in sagittal orientation with the following parameters: FOV = 230 × 226 mm^2^; slice thickness = 1.2 mm; acquisition matrix = 208 × 203; TR = 6.8 ms; TE = 3.1 ms; flip angle = 9°; number of signal averages = 1.

### Experimental Protocol

The stimuli consisted of video clips with a duration of 4 s that showed potentially harmful activities for the back (shovelling soil with a bent back, lifting a flowerpot with slightly bent back and vacuum cleaning under a coffee table with a bent back) and neutral activities (walking up and down the stairs and walking on even ground). The videos were recorded from a 3rd person perspective (Meier et al., 2015). These daily activities were selected from the short electronic version of the Photograph Series of Daily Activities (PHODA) that has established a fear hierarchy of daily activities based on ratings of perceived harmfulness. Further, the video clips have been validated in a previous fMRI study that demonstrated differential brain activity within the amygdala between high- and low-FOM, pain-free individuals (Meier et al., 2015). The video clips were displayed using MR-compatible goggles (Resonance Technology, Northridge, CA, USA) connected to a computer running Presentation^®^ software (Neurobehavioral Systems, Davis, CA, USA). Subjects were asked to carefully observe the video clips, which were shown in a pseudo-randomized order (no more than two identical consecutive trials). The fMRI session consisted of 30 trials, and the three harmful and neutral activities were each presented five times. Immediately after the observation of the video clips, participants were asked to rate the perceived harmfulness of the activity on a VAS. The VAS was anchored with the endpoints “not harmful at all” (0) and “extremely harmful” (10). All ratings were performed using a MR compatible track ball (Current Designs, Philadelphia, PA, USA) that moved the indicator on the VAS scale. The duration of the inter-stimulus interval (ISI, after the VAS rating, black screen with a green fixation cross) was jittered between 6 and 8 s.

### Image Pre-Processing

SPM12 (release 6470) was used for the brain activity analysis^1^. Functional EPI volumes of each subject were corrected for differences in head motion, spatially normalized according to the Montreal Neurological Institute (MNI) space and finally smoothed with a 8 mmfull-width at half-maximum (FWHM) Gaussian kernel.

### Brain Activity Analysis

To account for possible confounding head movement effects, individual movement parameters (translations in *x*, *y* and *z*-direction, as well as rotations around *x*, *y*, and *z* axis) were implemented in the 1st level model as regressors of no interest. Excessive head motion was defined as a dislocation of more than one in-plane voxel dimension (>2.5 mm). For removing low frequency noise, a high-pass filter with a cut-off of 128 s was used. Trials were modeled as boxcar regressors and convolved with the standard canonical hemodynamic response function (HRF) as implemented in SPM12.

In the first-level analysis, the general linear model (GLM) was fitted for each subject by a design matrix composed of the onsets and duration (4 s) of the harmful and neutral movement video clips (each pooled across the three different movement types resulting in 15 harmful and 15 neutral stimuli). For each subject, parameter estimates (beta) and contrast images (cons) were computed. In the subsequent statistical analyses only the contrast “harmful movements > neutral movements” was used. The resulting contrast images were analyzed using a random-effects model to allow for population inferences (Friston et al., 1999). For the second-level statistical inference, we conducted two different approaches. Similar to the methodology of Barke et al. (2012), we conducted a categorial analysis by dividing the cLBP group in high and low-FOM individuals using a median split (median = 37.5). In addition, we applied the same procedure to the pain-free group (median = 35) which resulted in four groups including 10 subjects, respectively. We then implemented the individual “harmful movements > neutral movements” contrasts in a factorial design with between-subject factors “FOM level” and “Group”. By means of this approach we aimed at identifying differential effects between groups by comparing means and looking at main and interaction effects. In a second approach we treated the TSK score as a continuous variable and performed a whole brain voxel-wise regression analysis by using one- (including all TSK scores) and two-sample *t*-test (including TSK scores for each group separately to test for interaction effects). The individual TSK scores were added within the GLM as covariates of interest without centering to investigate a positive or negative covariance with brain activity. Further, this allowed the identification of brain areas where there was a difference in activity between groups that varied as a function of the FOM (interaction contrasts “0 0 1 −1” and “0 0 −1 1”). Related correlation coefficients were calculated using the formula:

$$r\;=\;\sqrt{\frac{t^{2}}{t^{2}+\text{degrees of freedom}}}$$

using the maximum *t*-value of the respective cluster. The variance between groups was assumed to be unequal. Error covariance components were estimated using restricted maximum likelihood, as implemented in SPM12. To control for false positives, we used cluster-based family-wise error correction (FWE) based on the Gaussian Random Field Theory (Bennett et al., 2009). The clusters were identified by applying an initial uncorrected threshold of *p* < 0.001 and were considered to be significant if they fell below a cluster-corrected *p*(FWE) < 0.05. A initial threshold of *p* < 0.001 is recommended to avoid several pitfalls associated with cluster-based thresholding (Woo et al., 2014; Eklund et al., 2016). The resulting corrected SPM clusters were extracted with MarsBaR^2^, color-coded and finally superimposed onto the avg152T1-MNI brain using xjview^3^.

There is substantial *a priori* knowledge regarding the involvement of the amygdala in fear learning and expression. In addition, feeding the meta-analysis platform “neurosynth.org” (Yarkoni et al., 2011) that is based on reverse-inference maps with the term “fear” reveals strong evidence against the null hypothesis of no activation in the bilateral amygdala (up to *z*-scores of 13.0). As such, there is substantial evidence for a non-zero association between fear and amygdala activation across the population of published fMRI studies. Therefore, a small volume correction (SVC) on functionally derived masks of the amygdala was applied because whole-brain error correction is often too conservative when focusing on small brain regions such as the amygdala. SVC simply applies the FWE correction to a predefined volume which enhances the sensitivity of the results (Worsley et al., 1996). We used specific and study-independent 8 mmdiameter spherical masks of the amygdala that have been derived from computationally-modeled of fear- and reinforcement learning for pain (Zhang et al., 2016, peak MNI coordinates right amygdala: 27 −5 −10, left amygdala: −18 −2 −16). The SVC threshold was set to *p*(FWE) < 0.05 encompassing the voxel space of the bilateral amygdala. Beside the amygdala as a key region we focused also on other fear-related brain regions such as the insula, cingulate gyrus, fusiform gyrus, the orbitofrontal cortex (OFC), substantia nigra and the striatum (Etkin and Wager, 2007).

### Functional Connectivity Analysis

Functional connectivity was computed by using psycho-physiological-interactions (PPI) which provide a test of task effects on connectivity by assessing covariance between regions across time. Importantly, this covariance is assessed on the neural level, which results in a change in the BOLD signal, rather than at the level of BOLD signal, which is an indirect measure of neural activity. As such, a deconvolution step is necessary to assess the neural signal on which interaction analyses are performed (Kim and Horwitz, 2008; McLaren et al., 2012). Therefore, for each subject, we extracted the deconvolved time course averaged across a 6 mm-sphere around the peak voxel within the amygdala (MNI −14 0 −10) identified in the voxel-wise regression analysis (see “Whole Brain Voxel-Wise Regression Analysis” Section). Subsequently, separate psychological terms (harmful and neutral video clips), physiological regressors (time course of seed region) and PPI interaction terms, as well as the movement parameters, were included in a generalized PPI model. It is necessary to include all three time courses to exclude effects that are driven by a shared task input so that the variance explained by the interaction term shows only that over and above what is explained by the main effects of task and physiological correlation (O’Reilly et al., 2012). The generalized form of the context-dependent PPI approach increases the flexibility of the statistical modeling and improves single-subject model-fit, thereby increasing the sensitivity to true positive findings and a reduction in false positives (McLaren et al., 2012). The resulting PPI connectivity estimates (contrast harmful movements > neutral movements) were then taken again in a factorial design and in a whole-brain voxel-wise regression analysis as described in the brain activity analysis (see “Brain Activity Analysis” Section). Identified clusters were considered to be significant when falling below a cluster-corrected *p*(FWE) < 0.05, with an initial uncorrected threshold of *p* < 0.001.

## Results

### Questionnaires

None of the collected questionnaire data (STAI, FABQ, Bournemouth, pain measures) differed significantly between groups (Table 1).

### Stimulus Ratings

A repeated measures ANOVA for the ratings of the video clips with between-factors “Group” and “FOM Level” and within-factor “video type” yielded no main effects for “Group” and “FOM Level” (*F* < 0.8, *p* > 0.38), nor any interaction effects (*F* < 1.5, *p* > 0.24). However, a significant main effect for “video type” (*F*_(3,36)_ = 135.76, *p* < 0.001) could be observed. The potentially harmful activities were rated significantly more harmful than the neutral activities (Table 1).

### Brain Activity Results

#### Factorial Analysis (ANOVA)

**Figure 1:**
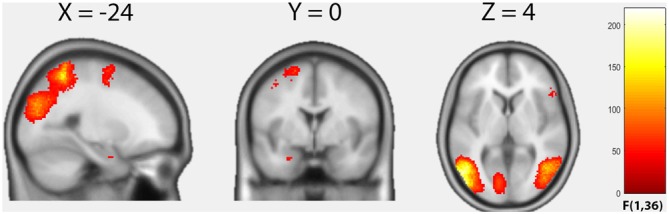
**Results of the factorial analysis.** Average effect of condition (*F*-contrast) “harmful movements > neutral movements”. Statistical maps are thresholded with *p* < 0.05, voxel-level family-wise error correction (FWE)-corrected.

We first examined the average effect of condition by means of an *F*-test to identify any effect on brain activity across the four groups induced by the harmful movements relative to the neutral movements (Figure 1, Table 2). Because in this contrast the results based on cluster inference revealed large clusters crossing multiple anatomical boundaries and therefore violated its theoretical rationale (Woo et al., 2014), we applied the more conservative voxel-based correction using a FWE-corrected threshold of *p* < 0.05. Relative to the neutral movements, the harmful movements induced significantly enhanced brain activity in the left middle temporal gyrus spanning in the left fusiform gyrus and the left inferior parietal cortex (cluster size = 9137, peak MNI: −54 −68 2, *F* = 218.63, *p*(FWE_voxel_) = 0.001), in the right middle occipital gyrus (cluster size = 2317, peak MNI: 48 −64 2, *F* = 142.25, *p*(FWE_voxel_) = 0.001), in the right superior parietal cortex (cluster size 927, peak MNI: 24 −54 62, *F* = 115.56, *p*(FWE_voxel_) = 0.001), in the left superior frontal gyrus (cluster size 306, peak MNI: −20 0 66, *F* = 78.09, *p*(FWE_voxel_) = 0.001), in the right inferior frontal gyrus (cluster size 79, peak MNI: 52 22 10, *F* = 77.88, *p*(FWE_voxel_) = 0.001) and in the left amygdala (cluster size 8, peak MNI: −26 0 −26, *F* = 77.88, *p*(FWE_voxel_) = 0.004).

**Table 2:** **Cluster maxima and their respective coordinates**.

Cluster size	*p*(FWE)	*F*	MNI coordinates (mm)			Brain region (AAL label)
			*x*	*y*	*z*	
**a. Factorial analysis (ANOVA), average effect of condition, contrast “harmful activites > neutral activities”**						
9137	0.001	218.63	−54	−68	2	Left middle temporal gyrus (Temporal_Mid_L)
2317	0.001	142.25	48	−64	2	Right middle temporal gyrus (Temporal_Mid_R)
927	0.001	115.56	24	−54	62	Right superior parietal cortex (Parietal_Sup_R)
306	0.001	78.09	−20	0	66	Left superior frontal gyrus (Frontal_Sup_L)
79	0.001	77.88	52	22	10	Right inferior frontal gyrus (Frontal_Inf_Tri_R)
8	0.004	50.48	−26	0	−26	Left amygdala (Amygdala_L)
**b. Voxel-wise regression analysis, positive correlations with TSK score, contrast “harmful activites > neutral activities”**						
349	0.001	6.04	−24	20	−22	Left orbitofrontal cortex (Fontral_inf_Orb_L)
194	0.001	5.82	−52	−32	−14	Left middle temporal gyrus (Temporal_Mid_L)
314	0.001	5.81	−4	−38	58	Left postcentral gyrus (Precuneus_L)
79	0.043	4.78	−58	−6	12	Left rolandic operculum (Rolandic_Oper_L)
339	0.001	4.64	−40	−56	34	Left angular gyrus (Angular_L)

*FWE, family-wise error; MNI, Montreal Neurological Institute; AAL, Automated Anatomical Labeling*.

Further, using *F*-contrasts, we examined the main effects “Group” and “FOM level” and their interaction based on the contrast “harmful movements > neutral movements”. No significant main effects for “Group” and “FOM level” could be detected, nor a significant interaction effect “Group × FOM level”. Within these contrasts, the additional SVC of the amygdala did not reveal any significant effects.

#### Whole Brain Voxel-Wise Regression Analysis

**Figure 2:**
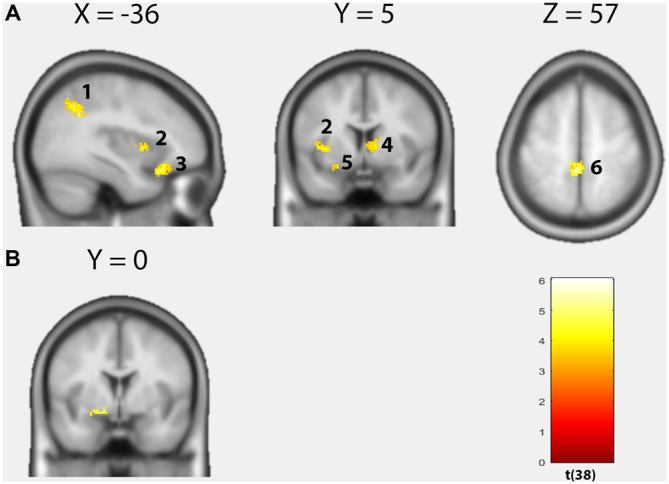
**Results of the voxel-wise regression analysis. (A)** Positive correlations between the Tampa Scale of Kinesiophobia (TSK) score and brain activity based on the contrast “harmful movements > neutral movements” (*p* < 0.05, cluster-level FWE-corrected). Left angular gyrus, left insula, left orbitofrontal cortex (OFC), right caudate, left amygdala, left postcentral gyrus/precuneus. **(B)** Significant group difference as a function of the TSK score in the left dorsal amygdala (Interaction 0 0 1 −1; *p* < 0.001, uncorrected).

Over both groups, the TSK score demonstrated an overall positive correlation with brain activity within seven distinct clusters (Figure 2A, Table 2). In the left OFC extending into the left amygdala (cluster size: 349, peak MNI: −24 20 −22, *t* = 6.04, *p*(FWE_cluster_) = 0.001, *r* = 0.70), in the left middle temporal gyrus (cluster size: 194, peak MNI: −52 −32 −14, *t* = 5.82, *p*(FWE_cluster_) = 0.001, *r* = 0.69), in the left postcentral gyrus (S1)/precuneus (cluster size: 314, peak MNI: −4 −38 58, *t* = 5.81, *p*(FWE_cluster_) = 0.001, *r* = 0.69), within the left rolandic operculum (cluster size: 79, peak MNI: −58 −6 12, *t* = 4.78, *p*(FWE_cluster_) = 0.043, *r* = 0.61), in the left angular gyrus (cluster size: 339, peak MNI: −40 −56 34, *t* = 4.64, *p*(FWE_cluster_) = 0.001, *r* = 0.58), in the left anterior insula (cluster size: 77, peak MNI: −42 6 4, *t* = 4.01, *p*(FWE_cluster_) = 0.001, *r* = 0.53) and in the right caudate (cluster size: 93, peak MNI: 12 2 8, *t* = 4.00, *p*(FWE_cluster_) = 0.020, *r* = 0.55). Furthermore, a significant group difference as a function of the TSK score (interaction 0 0 1 −1) could be detected in the left dorsal amygdala (cluster size: 6, peak MNI: −14 0 −10, *t* = 3.87, *p* < 0.001 uncorrected, SVC *p*(FWE_voxel_) < 0.05, Figure 2B). Feeding “neurosynth.org” with peak MNI coordinate [−14 0 −10] returned the highest *z*-score (11.61) for the term “amygdala”. No significant negative correlations with brain activity could be observed.

### Functional Connectivity Results (PPI Analysis)

#### Factorial Analysis (ANOVA)

Similar to the brain activity analysis, we first ran a categorial analysis including the four groups and used *F*-contrasts to examine the main effects “Group” and “FOM level” and their interaction based on the contrast “harmful movements > neutral movements” showing possible changes in functional connectivity using the left amygdala as a seed region (see “Whole Brain Voxel-Wise Regression Analysis” Section). No significant main effect for “Group” and “FOM level” could be observed, nor a significant interaction effect “Group × FOM level”.

#### Whole Brain Voxel-Wise Regression Analysis

**Figure 3:**
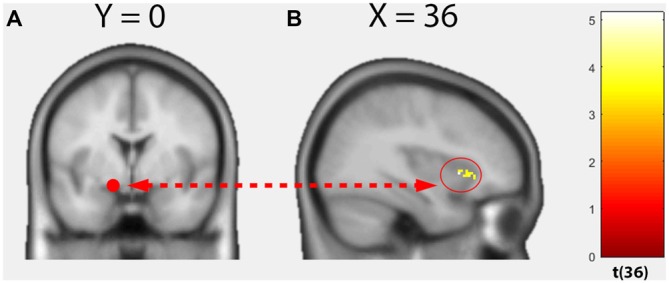
**Results of the voxel-wise regression psychophysiological-interactions (PPI) analysis. (A)** Seed region, left dorsal amygdala. **(B)** Functional connectivity group difference varied as a function of the TSK score between the seed region and right anterior insula (Interaction 0 0 1 −1; *p* < 0.05, cluster-level FWE-corrected).

Within groups, no significant positive correlations could be found between whole-brain functional connectivity of the left amygdala seed and TSK scores. However, a significant group difference dependent on the TSK score (interaction 0 0 1 −1) could be observed in the functional connectivity between the left amygdala seed and the right anterior insula (Figure 3, cluster size: 76, peak MNI: 36 16 0, *t* = 5.14, *p*(FWE_cluster_) = 0.011). No significant negative correlations could be detected.

## Discussion

The results of the current study are among the first to link brain activity within the fear circuit to a behavioral measure of FOM. Thus, current findings may contribute to the knowledge of underlying emotions described in the FA model by demonstrating that: (1) FOM positively correlated with activity in fear-related brain regions such as the insula and amygdala in both, cLBP patients and pain-free subjects; and (2) differential effects of FOM between pain-free subjects and cLBP patients were found in the extended amygdala and in its functional connectivity to the anterior insula.

FOM has substantial predictive power for perceived disability in cLBP and for its transition from a (sub-)acute pain state (Vlaeyen et al., 1995a,b; Vlaeyen and Linton, 2000; Houben et al., 2005; Cook et al., 2006; Wertli et al., 2014; Simons et al., 2015). The underlying FA model describes how FOM provokes avoidance behavior that gives rise to pain, disability, distress, and physical disuse which in turn results in hypervigilance for both pain and pain-related information (Vlaeyen et al., 1995a; Vlaeyen and Linton, 2000; Buer and Linton, 2002; Taylor et al., 2015). However, no neural substrates underlying back pain related FOM have been found to date and this therefore has initiated a debate about the precise contribution of fear in the FA model (Leeuw et al., 2007; Barke et al., 2012; Salomons and Davis, 2012). In the current study, we aimed to apply a novel approach to investigate neural correlates of FOM. First, we used video clips of potentially harmful activities for the back based on the fear hierarchy of PHODA. In a previous fMRI experiment, these video clips have been shown to elicit differential brain activity within the amygdala between high- and low-FOM in pain-free individuals (Meier et al., 2015). Second, the use of the TSK-G questionnaire permitted the measurement of FOM in the pain-free group and to compare high- and low-FOM individuals across the pain-free and cLBP groups. This allowed the implementation of two different statistical approaches: similar to Barke et al. (2012) we first conducted a 2 × 2 factorial analysis including high- and low-FOM individuals of both groups by using median splits. Except for an overall effect of activation in the left amygdala, which underlines the appropriateness of our FOM inducing stimuli, the results of the factorial analysis comparing means between groups were comparable to the results of Barke et al. (2012). No differential effect between groups could be detected in fear-related brain regions. Yet, there are obvious disadvantages when using a median split as a method of transforming a continuous variable into a categorical one. When a continuum is categorized, every value above the median, for example, is treated equally which also leads to a substantial loss of power (Aiken and West, 1991). Therefore, we treated the TSK score as a continuous variable and performed an additional whole-brain voxel-wise regression analysis to examine possible covariance of brain activity/connectivity with the strength of FOM. Indeed, the brain activity regression analysis revealed key brain regions associated with fear processing, namely the left OFC/amygdala and the left anterior insula. Interestingly, the right dorsal striatum which has been associated with aversive learning, memory processes and goal-directed behavior, also demonstrated a positive covariance with the TSK score (Delgado et al., 2008, 2009; Yanike and Ferrera, 2014). Furthermore, a significant difference as a function of FOM between pain-free and cLBP subjects could be observed in the left dorsal amygdala. Taking this area as a seed region and analyzing its functional connectivity across the whole brain, a further significant group difference as a function of FOM was observed in the connectivity to the right anterior insula.

Over both groups, FOM positively correlated with activation in a cluster of the left OFC extending into the left amygdala. The amygdala is a primary cortical source involved in bottom-up emotional processes including the evaluation and representation of perceived fear intensity/pain and contributes essentially to the deciphering of threats in visual scenes (Seifritz et al., 2003; Wager et al., 2004; Ellingsen et al., 2013; Kryklywy et al., 2013; Silvers et al., 2015). Furthermore, the amygdala constitutes an important site for a reciprocal relationship between persistent pain and negative affective states such as fear and anxiety (Neugebauer et al., 2004). Ergo, a stimulus that predicts an aversive outcome such as back pain might change its neural transmission in the amygdala to produce the somatic, autonomic and endocrine signs of fear, as well as increased attention to that stimulus in individuals with high FOM (Davis and Whalen, 2001). Furthermore, connections between the amygdala and OFC have been noted to be crucial for emotional learning. While the amygdala encodes the emotional consequences of events or actions, the OFC is important for adaptive changes in behavior as those consequences are experienced (Baxter et al., 2000; Pickens et al., 2003). Hence, the positive covariance of FOM severity with activation in the left OFC/amygdala cluster across both groups may reflect attempts of high FOM individuals to evaluate and/or regulate possible responses to the anticipated painful stimulus that are independent of a chronic pain condition and thus might be more driven by (pain-related) social fear learning (Olsson and Phelps, 2007). Support for this assumption comes from a previous study demonstrating a positive relationship of FOM severity and left amygdalar activation in pain-free individuals (Meier et al., 2015). However, indicative of a possible influence of chronic pain, FOM induced a significant and differential effect between pain-free and cLBP patients in a more dorsal cluster within the left amygdala. Feeding the meta-analysis platform “neurosynth.org” with the respective peak MNI coordinate (−14 0 −10) and searching after reports describing the same location within a 6 mm radius, studies refer to the ventral basal forebrain (VBF) including the bed nucleus of the stria terminalis (BNST; Davis and Whalen, 2001; Phan et al., 2003; Somerville et al., 2010). The BNST is considered as an extension of the central nucleus of the amygdala and is assigned to the “extended amygdala” (Alheid and Heimer, 1988). It has been proposed that activation in the VBF/BNST is more associated with sustained vigilance characterized by temporally extended changes in arousal compared to the neural processing of cued responses to discrete and immediate threats in the basolateral amygdala (Davis et al., 1997; Davis and Shi, 1999; Waddell et al., 2006; Somerville et al., 2010). cLBP patients demonstrated a positive and closer relationship of FOM and activation in this brain area compared to the pain-free group (interaction effect), suggesting a specific mechanism that might be related to hypervigilant monitoring of potentially harmful movements for the back due to fear/anxiety that is nurtured through the negative affectivity, recurrent pain and avoidance behavior in cLBP patients. Interestingly and in support of this view, a similar interaction effect could be detected in the functional connectivity between the VBF/BNST and the right anterior insula, revealed by the gPPI analysis. This fits well with studies reporting enhanced joint activation of the VBF/BNST and the insula that is related to the continuous hypervigilant monitoring of changes in environmental threat level in highly anxious individuals (Eysenck, 1992; Etkin and Wager, 2007; Somerville et al., 2010).

Although differences on a neuronal level were demonstrated, both groups rated the potential averseness of activities equivalently. In addition to a possible lack of sensitivity of the applied VAS scale, the absence of group differences on a behavioral level may indicate an unconscious neuronal process. This is in line with another study reporting no significant group differences in self-reported emotional arousal between healthy and chronic pain subjects suggesting that the differences observed are implicit, rather than explicitly reported (Simons et al., 2015).

Our findings may lead to a more mechanistic understanding on the neural level that supports preventative psychology-based interventions in acute episodes or *in vivo* exposure in individuals with elevated FOM. Furthermore, beside pain education for (sub-)acute and chronic LBP patients these results suggest a call for greater public education in terms of erroneous beliefs regarding back pain (Buchbinder et al., 2001, 2008; Engers et al., 2008). However, despite current cross-sectional study findings support the FOM component of the FA model, further studies based on long-term observations and appropriate psychological interventions are necessary to establish a causal relationship between the observed FOM related brain signatures and cLBP.

## Author Contributions

MLM: conception and design, analysis and interpretation of the data, drafting the work, final approval of the version and agreement to be accountable for all aspects of the work. PS: acquisition of the data, revising it critically for important intellectual content, final approval of the version and agreement to be accountable for all aspects of the work. AV: analysis and interpretation of the data, revising it critically for important intellectual content, final approval of the version and agreement to be accountable for all aspects of the work. BKH: conception and design, revising it critically for important intellectual content, final approval of the version and agreement to be accountable for all aspects of the work. ES: conception and design, revising it critically for important intellectual content, final approval of the version and agreement to be accountable for all aspects of the work. SH-B: conception and design, acquisition of the data, revising it critically for important intellectual content, final approval of the version and agreement to be accountable for all aspects of the work.

## Conflict of Interest Statement

The authors declare that the research was conducted in the absence of any commercial or financial relationships that could be construed as a potential conflict of interest.

## Footnotes

^1^http://www.fil.ion.ucl.ac.uk/spm/software/spm12/

^2^http://marsbar.sourceforge.net/

^3^http://www.alivelearn.net/xjview8/
